# No Influence of Positive Emotion on Orbitofrontal Reality Filtering: Relevance for Confabulation

**DOI:** 10.3389/fnbeh.2016.00098

**Published:** 2016-05-31

**Authors:** Maria Chiara Liverani, Aurélie L. Manuel, Adrian G. Guggisberg, Louis Nahum, Armin Schnider

**Affiliations:** Laboratory of Cognitive Neurorehabilitation, Division of Neurorehabilitation, Department of Clinical Neuroscience, University Hospital and University of GenevaGeneva, Switzerland

**Keywords:** reality monitoring, temporal consciousness, confabulation, orbitofrontal cortex

## Abstract

Orbitofrontal reality filtering (ORFi) is a mechanism that allows us to keep thought and behavior in phase with reality. Its failure induces reality confusion with confabulation and disorientation. Confabulations have been claimed to have a positive emotional bias, suggesting that they emanate from a tendency to embellish the situation of a handicap. Here we tested the influence of positive emotion on ORFi in healthy subjects using a paradigm validated in reality confusing patients and with a known electrophysiological signature, a frontal positivity at 200–300 ms after memory evocation. Subjects made two continuous recognition tasks (“two runs”), composed of the same set of neutral and positive pictures, but arranged in different order. In both runs, participants had to indicate picture repetitions within, and only within, the ongoing run. The first run measures learning and recognition. The second run, where all items are familiar, requires ORFi to avoid false positive responses. High-density evoked potentials were recorded from 19 healthy subjects during completion of the task. Performance was more accurate and faster on neutral than positive pictures in both runs and for all conditions. Evoked potential correlates of emotion and reality filtering occurred at 260–350 ms but dissociated in terms of amplitude and topography. In both runs, positive stimuli evoked a more negative frontal potential than neutral ones. In the second run, the frontal positivity characteristic of reality filtering was separately, and to the same degree, expressed for positive and neutral stimuli. We conclude that ORFi, the ability to place oneself correctly in time and space, is not influenced by emotional positivity of the processed material.

## Introduction

To sense whether a thought constitutes a souvenir from the past, a potential plan for the future, or refers to current reality is crucial for meaningful behavior. Failure to make this distinction is associated with reality confusion, as expressed in disorientation and confabulations, which patients act upon. We have called this syndrome, which corresponds to the original description of the Korsakoff syndrome (Korsakoff, 1891; Jolly, 1897), “behaviorally spontaneous confabulation” (Schnider, 2003, 2008).

Confabulations have received multiple interpretations as a combination of amnesia with executive dysfunction (Kapur and Coughlan, 1980; Papagno and Baddeley, 1997; Nys et al., 2004), a failure to “monitor” memory output (Burgess and Shallice, 1996; Moscovitch and Melo, 1997; Johnson and Raye, 1998; Gilboa et al., 2006), a disturbed sense of time (Talland, 1961; Dalla Barba, 1993), or a desire to fill gaps in memory to avoid embarrassment (Flament, 1957) and maintain self-coherence (Conway and Tacchi, 1996). A related proposal holds that motivational factors may induce a positively biased memory recall in amnesia, which would lead to confabulations (Fotopoulou et al., 2007, 2008; Fotopoulou, 2010). Fotopoulou et al. (2008) based this proposal on the observation of a positive emotional bias in the content of confabulations. Similarly, Alkathiri et al. (2015) described a positive bias in the false recall and recognition of confabulating patients.

These hypotheses tried to explain confabulations as a purely verbal phenomenon, irrespective of the presence of reality confusion. We found that patients who confabulate in the context of reality confusion, as reflected in acts according to the confabulations and disorientation, specifically failed in an experimental task. They underwent repeated runs of a continuous recognition task, each composed of the same set of pictures but arranged in different order, and had to indicate whether for each picture they had seen it in the current run. Healthy subjects usually have no difficulty in distinguishing between pictures seen in previous or in the current run. In contrast, patients with behaviorally spontaneous confabulations had a specific increase of false positives from the second run on (Schnider et al., 1996a,b; Schnider and Ptak, 1999; Nahum et al., 2012). Recovery from reality confusion was accompanied by normalization of the false positive rate (Schnider et al., 2000a). In reality confusing patients, lesions typically concern the posterior medial orbitofrontal cortex or directly connected structures (Schnider et al., 1996a; Schnider and Ptak, 1999). Conversely, healthy subjects correctly performing the task, on which the patients had failed, activated the posterior medial orbitofrontal cortex, area 13 (Schnider et al., 2000b). We have called the ability measured with this task orbitofrontal reality filtering (ORFi; Schnider, 2013). Event-related potentials (ERPs) in healthy subjects performing the task showed a distinct frontal positivity at about 200–300 ms in response to the stimuli on which the patients had failed, namely, first appearances (“distracters”) within the second run (Schnider et al., 2002; Wahlen et al., 2011; Liverani et al., 2015).

Patients with behaviorally spontaneous confabulation typically enact common habits: they think they have business meetings or familial obligations. There is no obvious positive emotional bias in their percept of reality, which is at odds with the claim that confabulations do have such a bias. If Fotopoulou et al. (2008) hypothesis also applied to behaviorally spontaneous confabulation, then ORFi would be expected to be influenced by the emotional valence of stimuli, too. In the present study we investigated whether ORFi is indeed influenced by positive emotion.

We composed two runs of a continuous recognition task, similar to previous clinical and imaging studies (Schnider, 2003, 2008), but including both neutral and emotionally positive pictures. We expected that there would be a valence effect in the ERP’s from about 200–300 ms on (Palomba et al., 1997; Cuthbert et al., 2000; Maratos et al., 2000; Bradley et al., 2007; Van Strien et al., 2009). Critical for the present study, however, we expected that stimuli’s emotion would have no influence on reality filtering: there would be the typical frontal positivity in response to first appearances of pictures in the second run around 200–300 ms irrespective of or superimposed on emotional valence (Schnider et al., 2002; Wahlen et al., 2011; Liverani et al., 2015).

## Materials and Methods

### Participants

Twenty right-handed participants gave written informed consent and were paid to participate in the study. They reported no history of psychiatric or neurological disorders or medication use. One subject was excluded from the study because of poor signal quality of electrophysiological data. Finally 19 subjects (10 females, age 27 ± 5 years) were included in the analysis. The research was approved by the local Ethics Committee and conducted according to the Declaration of Helsinki.

### Stimuli

The set of positive and neutral stimuli consisted of 240 realistic, high-quality photographs from the Nencki Affective Picture System (NAPS; Marchewka et al., 2014), divided into five categories: landscapes; objects; animals; people and faces. The NAPS was chosen as it allows one to match pictures according to visual features, such as complexity, luminosity and contrast, which are critical for an ERP study. Pictures were selected on the basis of their original valence rating, which was collected using a bipolar scale ranging from 1 to 9 (with 1 = *very negative*, 5 = *neutral*, 9 = *very positive*; Marchewka et al., 2014). Half of the pictures had a neutral valence (4.7–5.3; mean = 5 ± 0.2), the other half had a positive valence (7.3–8.5; mean = 7.7 ± 0.3; *t*_(119)_ = 273, *p* < 0.001). Positive pictures were also more relaxing: they had lower arousal ratings (2–4.5; mean = 3.8 ± 0.9) than neutral pictures (4.1–7.4; mean = 5.2 ± 0.6; *t*_(119)_ = 14.65, *p* < 0.001). Stimuli were matched with respect to their luminance, contrast and entropy (*t*_(119)_ > 172, *p* > 0.65).

### Task Composition

**Figure 1:**
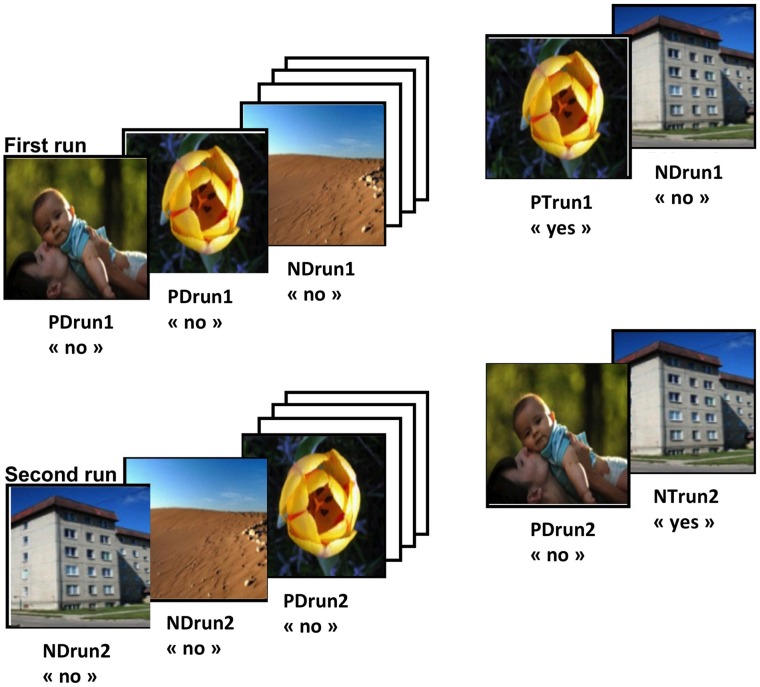
**Task design.** Each block was composed of two runs. Positive Distracters (PD) and Neutral Distracters (ND) are positive and neutral images that are presented for the first time within a run, respectively. Positive Targets (PT) and Neutral Targets (NT) are positive and neutral images that are repeated within the same runs, respectively. Pictures from the Nencki Affective Picture System (Marchewka et al., 2014), reprinted with permission. For abbreviations see Table 1.

To avoid fatigue, subjects performed three independent blocks of the task, separated by a 10-min′ break. Each block was constituted of a different set of 80 images (40 positive, 40 neutral) used to compose two runs of a continuous recognition task (Figure 1). The two runs of a block were composed of the same pictures. Picture repetitions occurred after 8–16 intervening images. Participants were asked to indicate picture repetitions within, and only within, the currently ongoing run by pressing the right button with the right middle finger to indicate first presentations (distracters; “No, not yet seen”) within the run and the left button with the right index for repeated images within the ongoing run (targets; “Yes, already seen.”). The first run measures learning and recognition and can be solved on the basis of familiarity alone. In the present study, this run thus contained the following stimulus types: positive distracters (PD), PDrun1; neutral distracters (ND), NDrun1; positive targets (PT), PTrun1; neutral targets, NTrun1.

The second run was used to test reality filtering (Schnider, 2008, 2013). The same set of images was presented, but rearranged in a different order. As subjects are already familiar with all stimuli, this run requires the ability to sense whether familiarity emanates from previous occurrence within the ongoing run (“ongoing reality”) or from the previous run. This run contains the following stimulus types: positive distracters, PDrun2; neutral distracters, NDrun2; positive targets, PTrun2; neutral targets, NTrun2.

Amnesic subjects typically have similar difficulty with this task’s first and second run (Schnider et al., 1996a; Schnider and Ptak, 1999); correct performance by healthy subjects activates the medial temporal lobe (Schnider et al., 2000b). Reality-confusing patients with confabulations and disorientation have a performance drop in the second run: they typically have a specific increase of false positives (Schnider and Ptak, 1999; Nahum et al., 2012). Performing this task’s second run activates the posterior orbitofrontal cortex in healthy subjects (Schnider et al., 2000b). Thus, the processing of distracters (PDrun2; NDrun2) is the critical capacity in the second run.

The two runs within each block were made in immediate succession. Images were presented on a 17″ computer screen for 2000 ms followed by an interstimulus interval with a fixation cross in the center of the screen presented for 700 ms. The task was performed using E-prime 2.0 (Psychology Software Tools, Inc., Pittsburgh, PA, USA^1^). Before the task, participants performed a training session with geometrical figures (*N* = 10) instead of the experimental pictures, in order to familiarize with task instructions.

### EEG Acquisition and Raw Data Processing

Electroencephalography (EEG) was continuously recorded with a 128-channel Active-Two Biosemi EEG system (Biosemi V.O.F. Amsterdam, Netherlands). Signal was sampled at 512 Hz and filtered at bandwidth of 0–104 Hz. Electrodes impedance was kept below 20 KΩ. EEG data preprocessing and analyses were performed with the Cartool Software^2^ developed by Brunet et al. (2011). Epochs from 100 ms before to 800 ms after the stimulus onset were averaged for each subject and each condition to calculate the event-related potentials (ERPs). ERPs were band-pass filtered to 1–30 Hz and recalculated against the average reference. Baseline correction was applied on the 100 ms pre-stimulus period. Only correct trials were retained in the analysis and epochs with artifacts higher than 100 μV were automatically excluded. In addition, data were visually inspected and EEG epochs with eye blinks, movements or other sources of transient noise were rejected. Before the group averaging, channels with substantial noise were interpolated using a spherical spline interpolation (mean 4.2% of interpolated electrodes; Perrin et al., 1987). The epoch from 0 to 800 ms after stimulus onset was retained for analysis. The mean number (± SD) of accepted epochs was 73 ± 20.8 for PDrun1, 75.5 ± 21 for NDrun1, 74.7 ± 20.4 for PTrun1, 74.3 ± 21.2 NTrun1, 72.9 ± 23 for PDrun2, 71.4 ± 25.1 for NDrun2, 68.8 ± 25.7 for PTrun2 and 70.7 ± 27.4 for NTrun2. 2 × 2 × 2 repeated measures analysis of variance (ANOVA) with factors Run (1,2), Stimulus (Distracter, Target) and Emotion (Positive, Neutral) revealed no statistical differences in number of epochs (*p* > 0.05).

### Behavioral Data Analyses

The 2 × 2 × 2 repeated measures ANOVAs on percentage of correct responses and reaction time were performed with the within-subjects factors Run (1,2), Stimulus (D,T) and Emotion (P,N). When appropriate, *post hoc* Fisher’s tests were performed, with a significance level of *p* < 0.05.

### Topographic Patterns Analyses (TANOVA)

To identify periods with significant statistical differences in the topography of the electric field across conditions, a time-wise 2 × 2 topographic ANOVA (TANOVA) was performed for each run. This is a non-parametric randomization test (5000 randomizations per time point) based on global dissimilarities between electric fields implemented in the RAGU Software (Randomization Graphical User Interface; Koenig et al., 2011). Analysis was performed using a within-subject design with the factors Stimulus (D,T) and Emotion (P,N). The statistically significant periods of interest were defined by a *p* < 0.05 for ≥20 ms (Guthrie and Buchwald, 1991; See Murray et al., 2006; Toepel et al., 2014; Manuel and Schnider, 2016, for a similar procedure).

### Global Waveform Analyses

Electrode- and time-wise 2 × 2 repeated measures ANOVAs on the ERP waveform for each of the 128 electrodes were conducted with the factors Stimulus (D,T) and Emotion (P,N) for each run. This analysis was performed with the Statistical Toolbox for Electrical Neuroimaging (STEN) developed by Jean-François Knebel^3^. To account for temporal autocorrelation only periods that remained significant (*p* < 0.05) for ≥20 ms were considered reliable. We also applied a spatial extent criterion of at least five electrodes (i.e., 4% of the electrode montage) for each time sample considered statistically significant.

### Regional Frontal Waveform Analysis

Since the electrophysiological correlate of ORFi is typically expressed in the frontal region (Schnider et al., 2002), we defined a region of interest (ROI) composed of 16 electrodes in the frontal part of the scalp (Dien and Santuzzi, 2005). ERPs of electrodes in this region were averaged for each condition and each subject. Apparent differences were statistically tested using repeated-measures ANOVAs with the factors Stimulus (D,T) and Emotion (P,N) for each run and subjected to *post hoc* tests.

## Results

### Behavioral Results

Behavioral results are summarized in Table 1. Accuracy was better for neutral than positive pictures (*F*_(1,18)_ = 14.56, *p* = 0.001, $\eta_{p}^{2}$ = 0.45). There was a significant interaction Run × Stimulus (*F*_(1,18)_ = 9.93, *p* = 0.006, $\eta_{p}^{2}$ = 0.36). *Post hoc* tests revealed that accuracy was better in response to Distracters than Targets in the first, but not the second run (*t*_(18)_ = 1.73, *p* = 0.01). Reaction times were longer in run 2 than run 1 (*F*_(1,18)_ = 14.60, *p* = 0.001, $\eta_{p}^{2}$ = 0.45), in response to Distracters than Targets (*F*_(1,18)_ = 17.56, *p* = 0.001, $\eta_{p}^{2}$ = 0.49) and in response to positive than neutral pictures (*F*_(1,18)_ = 17.49, *p* = 0.001, $\eta_{p}^{2}$ = 0.49).

**Table 1:** **Behavioral results**.

Stimulus type	%Correct responses	Reaction times (ms)
PDrun1	98.0 ± 2.6	809.6 ± 222.3
NDrun1	99.0 ± 2.3	786.9 ± 203.8
PTrun1	94.2 ± 6.5	732.7 ± 85
NTrun1	96.3 ± 4.0	727.6 ± 85.9
PDrun2	96.2 ± 3.6	835.2 ± 249.2
NDrun2	96.7 ± 2.8	829.8 ± 237.5
PTrun2	95.2 ± 5.2	767.7 ± 89.3
NTrun2	96.2 ± 4.4	747.7 ± 83.3

*The second column indicates the percentage of correct responses ± standard deviation (SD) for each type of stimulus. The third column indicates mean of reaction times ± SD in ms for each type of stimulus. PDrun1, positive distracters of run 1; NDrun1, neutral distracters of run 1; PTrun1, positive targets of run 1; NTrun1, neutral targets of run 1; PDrun2, positive distracters of run 2; NDrun2, neutral distracters of run 2; PTrun2, positive targets of run 2; NTrun2, neutral targets of run 2*.

### Topographic Pattern Analysis (TANOVA)

**Figure 2:**
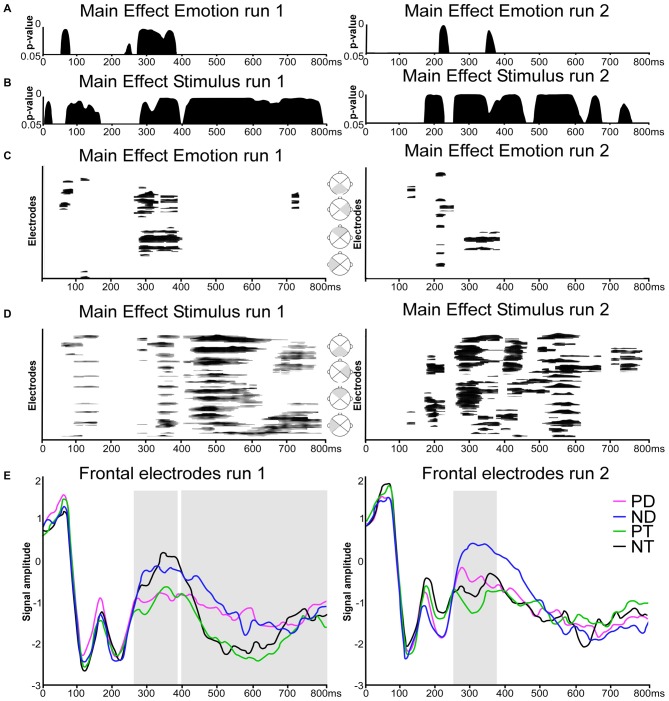
**Electrical neuroimaging results. (A,B)** Time-wise topographic analysis on run 1 and 2. Results of the 2 × 2 repeated measures analysis of variances (ANOVAs) on the topography with the factors Stimulus (Distracters, Targets) and Emotion (Positive, Neutral). **(C,D)** Electrode- and time-wise ERP waveform analysis on run 1 and 2 and on different scalp regions (gray triangles). Results from the 2 × 2 repeated measures ANOVAs on the waveforms with the factors Stimulus (Distracters, Targets) and Emotion (Positive, Neutral). Black lines indicate significant effects with *p* < 0.05 and lasting at least 20 ms. **(E)** Grand average ERPs waveforms for the 16-electrodes frontal ROI in response to (PD, in magenta), Neutral Distracters (ND, in blue), Positive Targets (PT, in green) and Neutral Targets (NT, in black). Traces are displayed in microvolts (μV) as a function of time relative to stimulus onset. Repeated measures ANOVA were calculated across the time window derived from topographic patterns analyses (TANOVA) and boxed in gray (run 1: 270–390 and 400–800 ms; run2: 250–380 ms).

The TANOVA revealed significant effects of Emotion in both runs, but more temporally consistent in run 1, in a relatively early period, between about 270 and 390 ms (Figure 2A). The effects of Stimulus type (Figure 2B) were more extended in both runs but with distinct differences. Specifically, significant topographic differences were observed in the following periods:

#### Run 1

There was an effect of Emotion at about 270–390 ms post stimulus onset (Figure 2A, left column). Stimulus types induced an early main effect at about 70–170 ms, and a later, prolonged effect from 280 ms on (Figure 2B, left column).

#### Run 2

There was a main effect of Emotion at 210–240 ms and a brief effect at 350–370 ms (Figure 2A, right column). A main effect of Stimulus was present at 170–230 ms, and from 250 ms on (Figure 2B, right column).

### Global Waveform Analysis

Global waveform analysis using ANOVAs over all electrodes yielded results consistent with the TANOVA (Figures 2C,D): emotion was expressed in both run, but more distinctly in run 1, mainly at about 280–400 ms, with brief effects before and after. Stimulus type had extended main effects, from about 350–800 ms in the first run. In the second run, extended effects were present at an earlier stage, at around 200 and from about 280–350 ms. In details, there were the following effects:

#### Run1

As visible in Figure 2C (left column), ANOVAs over all electrodes showed an effect of emotion at 45–80 ms, 105–130 ms, 260–400 ms, 720–740 ms over frontal and lateral right scalp regions. There was a main effect of Stimulus over extended periods and extended scalp regions at 50–160 ms, 270–390 ms and 400–800 ms (Figure 2D, left column).

#### Run2

There was a significant main effect of Emotion at 120–140 ms, 200–255 ms and 290–390 ms (Figure 2C, right column), and a main effect of Stimulus at 165–220 ms, 250–380 ms, 390–460 ms, 490–670 ms, and 700–790 ms (Figure 2D, right column).

### Regional Frontal Waveform Analysis

As Figure 2E shows, Emotion was expressed in both runs at around 270–400 ms. In the first run, this effect prevailed over the effect of Stimulus type. In the second run, the effect of Stimulus type was superimposed on the effect of Emotion, in both cases with Distracters being less negative than targets. This corresponds to the expected signature of ORFi. After 400 ms, there was only an extended effect of stimulus type, but no effect of Emotion, consistent with the TANOVA and the Global Waveform Analysis. In detail, the following findings were obtained:

#### Run 1

A directed 2 × 2 repeated measures ANOVA on amplitude difference in the frontal ROI during the period showing the main effect of Emotion and of Stimulus (270–390 ms) in the TANOVA and waveforms results showed that neutral stimuli (both distracters and targets) evoked more positive responses than positive stimuli (*F*_(1,18)_ = 13.42, *p* = 0.002, $\eta_{p}^{2}$ = 0.43).

The late main effect of Stimulus present in both TANOVA and waveform analysis at about 400–800 ms corresponded to a higher positive potential for Distracters compared to Targets in the frontal region (*F*_(1,18)_ = 4.71, *p* = 0.04, $\eta_{p}^{2}$ = 0.21).

#### Run 2

There was a main effect of Stimulus at 250–380 ms: neutral stimuli evoked more positive responses than positive stimuli (*F*_(1,18)_ = 7.06, *p* = 0.02, $\eta_{p}^{2}$ = 0.28). In both cases, distracters evoked more positive responses than targets (*F*_(1,18)_ = 8.41, *p* = 0.02, $\eta_{p}^{2}$ = 0.29), similar to earlier studies on ORFi (Schnider et al., 2000b; Wahlen et al., 2011; Bouzerda-Wahlen et al., 2015; Liverani et al., 2015). There was no significant interaction (*p* = 0.127).

## Discussion

This study shows that ORFi is not dependent on or influenced by positive emotional valence of the processed information. The experiment used to test this is a confirmed surrogate marker of reality confusion in confabulating patients (Schnider et al., 1996a,b, 2000a; Schnider and Ptak, 1999; Gilboa et al., 2006; Nahum et al., 2012). It activates the orbitofrontal cortex (area 13) in healthy subjects (Schnider et al., 2000b; Treyer et al., 2003, 2006) and has a known electrophysiological signature: a relative frontal positivity (or absence of negativity) at about 200–300 ms in response to distracters of run 2 (Schnider et al., 2002; Wahlen et al., 2011; Bouzerda-Wahlen et al., 2015; Liverani et al., 2015).

In the present study, positive stimuli clearly differed from neutral ones: they were less well recognized in both runs of our task and they induced a more negative evoked potential over the frontal electrode in both runs around 250–400 ms. The behavioral result, albeit in contradiction to our hypothesis, is compatible with previous observations: emotional stimuli are typically better recognized after a delay (Palomba et al., 1997; La Bar and Phelps, 1998; Ochsner, 2000; Versace et al., 2010), but they may have no advantage (Van Strien et al., 2009; Treese et al., 2010) or even be less well recognized, typically due to more false positive responses, with short delays (Maratos et al., 2000; Windmann and Chmielewski, 2008). Another possible explanation lies in the lower arousal associated with the positive stimuli used—the price for truly “positive” pictures, which may have a relaxing effect. However, similar to valence, the behavioral effect of arousal is difficult to predict: while better retained at long intervals, high arousal stimuli may be less well recognized at short intervals (Kleinsmith and Kaplan, 1963). For the sake of the present study, it is important to note that the emotional stimuli did induce a different behavioral response than the neutral ones.

Similar considerations hold for the evoked potential response. Effects of emotionality have strongly varied across studies depending on the type and presentation of material and the specific task (Olofsson et al., 2008). In most studies, emotional stimuli (positive or negative) evoked more positive responses than neutral ones (Palomba et al., 1997; Cuthbert et al., 2000; Maratos et al., 2000; Bradley et al., 2007) at various time points and over different electrodes. Although results between studies differed considerably, it appears that valence effects (positive—negative) are typically expressed at an earlier stage, around 200–300 ms, than arousal effects, which occur at 300–1000 ms (Van Strien et al., 2009; Versace et al., 2010). The main potential differences in the present study occurred in the earlier period, around 250–400 ms, and may thus reflect the influence of the positive valence rather than the lower arousal of our emotional stimuli.

The present study was interested in the influence of positive emotion on ORFi and therefore, in the time period between 200–400 ms. Thus, the crucial result is that positive stimuli differed both behaviorally and electrophysiologically from neutral stimuli already when ORFi was not yet challenged, that is, in the first run. However, while the difference between positive and neutral stimuli also came out in the critical second run, the signature of reality filtering was present irrespective of the emotional valence of the stimuli. Thus, reality filtering worked on neutral and emotionally positive stimuli in the same way.

What does this result mean for the mechanism of reality confusion and confabulation? First, it indicates that the mechanism, whose failure induces reality confusion (Schnider et al., 1996a,b; Schnider and Ptak, 1999; Nahum et al., 2012), is not modulated by emotion. This is compatible with an additional line of evidence. As we have previously shown, the tendency of reality-confusing patients to enact daily routines appears to reflect an inability to abandon anticipations that are not currently valid. In other words, they have a specific defect of extinction capacity (Nahum et al., 2009, 2012). The typical signature of this need to abandon a behavior—a frontal positivity, which also occurs at about 200–300 ms (Schnider et al., 2007)- does not depend on whether the presented outcome is positive (reward) or neutral (no reward), but on the need to adapt behavior (Nahum et al., 2011).

In this study, we did not explore whether negative emotion would influence ORFi because there is no clinical evidence suggesting such a link. While potentials evoked by negative stimuli typically differ from positive stimuli (Van Strien et al., 2009), we would expect that ORFi would be expressed independently of negative emotion, similar to the present study.

Our result refers to a specific mechanism of reality confusion and confabulation—ORFi. It leaves open the possibility that motivational factors, such as positive emotions, influence the content of confabulations and maybe also the desire to verbally express them. Such factors have long been speculated to influence confabulations occurring in the context of amnesia (Flament, 1957; Weinstein, 1987; Fotopoulou et al., 2008), but also in other, non-cerebral disease states, “to explain away manifestations of illness” (Weinstein and Kahn, 1955). This notion is difficult to apply to confabulations emanating from reality confusion, all the more so because profoundly negative enactments have been described, such as, the repeated urge to organize the funeral of (still alive) loved ones (Korsakoff, 1891; Nahum et al., 2012).

In conclusion, ORFi, a mechanism necessary to keep thought and behavior in phase with ongoing reality, does not appear to be modulated by positive emotional valence of the evoked memories.

## Author Contributions

MCL: conception of the research, design of the experiment, subjects’ recruitment, data acquisition, data analysis, writing of the article. ALM: data acquisition, data analysis, writing of the article, revision of the article. LN: conception of the research, design of the experiment, data analysis, revision of the article. AGG: conception of the research, design of the experiment, data acquisition, revision of the article. AS: conception of the research, design of the experiment, data analysis, writing of the article, revision of the article.

## Funding

This work was supported by the Swiss National Science Foundation, Grant No. 32003B-155947 to AS.

## Conflict of Interest Statement

The authors declare that the research was conducted in the absence of any commercial or financial relationships that could be construed as a potential conflict of interest.

## Footnotes

^1^www.pstnet.com/eprime

^2^https://sites.google.com/site/fbmlab/cartool

^3^http://www.unil.ch/line/home/menuinst/about-the-line/software–analysis-tools.html

## Abbreviations

ERP: Event related potential
ND: Neutral distracter
NT: Neutral target
ORFi: Orbitofrontal reality filtering
OFC: Orbitofrontal cortex
PD: Positive distracter
PT: Positive target
TANOVA: Topographic ANOVA.
